# Dietary Polyphenols from *Dendropanax morbifera* Leaves Ameliorate Alcohol-Induced Liver Injury Through Regulation of Oxidative Stress and Nrf2 Signaling

**DOI:** 10.3390/nu18121902

**Published:** 2026-06-12

**Authors:** TaeKil Eom, Ju-Sung Kim

**Affiliations:** 1 Department of Herbology, College of Korean Medicine, Woosuk University, Jeonju 55338, Republic of Korea; taekil7@hanmail.net; 2 Department of Plant Resource and Environment, College of Agriculture & Life Sciences, SARI, Jeju National University, Jeju 63243, Republic of Korea

**Keywords:** alcohol liver disease, CYP2E1, Nrf2, antioxidant enzymes, *Dendropanax morbifera*

## Abstract

**Background/Objectives:** Excessive alcohol consumption induces hepatic injury primarily through cytochrome P450 2E1 (CYP2E1)-mediated reactive oxygen species (ROS) generation and disruption of redox homeostasis. This study investigated the hepatoprotective effects of the ethyl acetate fraction from *Dendropanax morbifera* leaves (DMLEAF) against ethanol-induced oxidative damage in vitro and in vivo. **Methods:** The protective mechanisms of DMLEAF were evaluated in HepG2 cells exposed to ethanol (400 mM, 24 h) and in an acute ethanol-induced liver injury mouse model. Cellular ROS levels, apoptosis, antioxidant enzyme activities, and nuclear factor erythroid 2-related factor 2 (Nrf2) translocation were assessed in vitro. Serum biochemical markers, histopathological changes, and hepatic CYP2E1 mRNA expression were analyzed in vivo. **Results:** In HepG2 cells, DMLEAF significantly reduced intracellular ROS levels and apoptosis, improving cell viability by up to 27.2% and reducing apoptosis by approximately 32%. DMLEAF also attenuated the ethanol-induced decrease in antioxidant enzyme activities (superoxide dismutase, catalase, glutathione peroxidase, and glutathione reductase) and promoted nuclear translocation of Nrf2. In mice, oral administration of DMLEAF significantly reduced serum alanine aminotransferase and aspartate aminotransferase levels, improved histopathological alterations, and suppressed hepatic CYP2E1 mRNA expression by 2.6-fold compared with ethanol-treated controls, while preventing the reduction in hepatic antioxidant enzyme activities. **Conclusions:** These findings suggest that DMLEAF mitigates alcohol-induced liver injury through suppression of CYP2E1-associated ROS production and activation of Nrf2-mediated antioxidant defense mechanisms.

## 1. Introduction

Alcohol consumption is a major global health concern and a leading cause of chronic liver disease. Excessive alcohol intake is closely associated with the development of alcoholic liver disease (ALD), a progressive condition ranging from steatosis to cirrhosis and hepatocellular carcinoma [1,2]. The global incidence and mortality of ALD continue to rise in parallel with increasing alcohol consumption. The liver plays a central role in alcohol metabolism, primarily through alcohol dehydrogenase (ADH) and aldehyde dehydrogenase (ALDH), which convert ethanol to acetaldehyde and subsequently to acetate [3]. However, chronic or excessive alcohol intake also induces CYP2E1, a key enzyme that metabolizes ethanol and generates excessive ROS as by-products [4,5]. Elevated CYP2E1 expression leads to oxidative stress, lipid peroxidation, and depletion of endogenous antioxidant defenses, ultimately contributing to hepatocellular injury and progression of ALD [6].

Oxidative stress is a critical driver of alcohol-induced liver injury. Among the endogenous protective mechanisms against oxidative damage, nuclear factor erythroid 2-related factor 2 (Nrf2) plays a pivotal role [7,8]. Under basal conditions, Nrf2 is sequestered in the cytoplasm by its inhibitory protein Kelch-like ECH-associated protein 1 (Keap1). Upon oxidative or electrophilic stress, Nrf2 dissociates from Keap1 and trans locates to the nucleus, where it binds to antioxidant response elements (AREs) and induces transcription of cytoprotective genes. These include heme oxygenase-1 (HO-1), NAD(P)H quinone oxidoreductase 1 (NQO1), glutathione peroxidase (GPx), glutathione transferase (GST), superoxide dismutase (SOD), catalase (CAT), and other phase II detoxifying enzymes [9,10]. Activation of the Nrf2 signaling pathway has therefore emerged as a promising therapeutic strategy for mitigating oxidative liver injury [11].

Growing evidence suggests that naturally occurring phytochemicals exert antioxidant and hepatoprotective effects through modulation of redox-sensitive signaling pathways, including activation of Nrf2 [12,13]. Polyphenolic compounds, in particular, have attracted attention due to their strong radical-scavenging capacity and ability to regulate intracellular antioxidant responses [14]. Several studies have reported that plant-derived polyphenols attenuate alcohol-induced oxidative stress by suppressing CYP2E1 expression and enhancing Nrf2-mediated antioxidant enzyme expression [15,16]. However, identification of bioactive fractions with defined phytochemical composition and elucidation of their precise molecular mechanisms remain areas of active investigation. Crude plant extracts contain a complex mixture of compounds with varying polarity, which may result in dilution or masking of bioactive constituents. Solvent fractionation enables the separation of these compounds based on their physicochemical properties, potentially leading to distinct biological activities among fractions. Ethyl acetate is known to preferentially extract moderately polar compounds, including certain phenolic constituents, which have been associated with antioxidant activity. Therefore, fractionation may yield fractions with differential biological effects compared with crude extracts.

*Dendropanax morbifera*, a member of the Araliaceae family native to Korea, Japan, and China, has traditionally been used as a medicinal plant [17]. Although other Araliaceae species such as ginseng have been extensively studied, the pharmacological properties of *D. morbifera* have only recently gained scientific attention [18,19]. Emerging studies indicate that *D. morbifera* leaves are rich in polyphenolic compounds, including chlorogenic acid and rutin, and exhibit potent antioxidant and anti-inflammatory activities [20,21]. These findings suggest that *D. morbifera* may represent a valuable source of natural antioxidants capable of modulating oxidative stress-related disorders.

In our previous study, we demonstrated that crude extracts of *D. morbifera* ameliorated alcohol-induced liver injury in association with modulation of the gut microbiota [22,23]. While these findings highlight the potential contribution of microbiota-related mechanisms, whether a specific solvent fraction directly regulates intracellular redox signaling pathways remains unclear. Given that ethanol-induced hepatotoxicity is largely driven by CYP2E1-mediated oxidative stress, investigation of specific fractions targeting this pathway is of particular relevance. Therefore, the objective of the present study was to identify a bioactive ethyl acetate fraction of *D. morbifera* leaves and to investigate its hepatoprotective effects against alcohol-induced liver injury through modulation of CYP2E1-mediated oxidative stress and Nrf2-dependent antioxidant signaling. To achieve this, phytochemical profiling was performed together with mechanistic evaluation in HepG2 cells and an acute ethanol-induced liver injury mouse model.

## 2. Materials and Methods

### 2.1. Materials

The human hepatocellular carcinoma cell line HepG2 was obtained from the American Type Culture Collection (ATCC, Manassas, VA, USA). Dulbecco’s modified Eagle’s medium (DMEM), fetal bovine serum (FBS), penicillin–streptomycin solution, and L-glutamine were purchased from Wellgene (Daegu, Korea). All primary antibodies used for Western blot analysis were obtained from Santa Cruz Biotechnology (Santa Cruz, CA, USA). All other chemicals were of analytical grade.

### 2.2. Preparation of Solvent Fraction from Dendropanax morbifera Leaves

The crude ethanol extract of *Dendropanax morbifera* leaves (DMLEE) was prepared as previously described [22]. Briefly, dried leaves were extracted with distilled water/ethanol (3:7, *v*/*v*), and the extract was filtered, concentrated under reduced pressure, and lyophilized. The obtained DMLEE was suspended in distilled water/methanol (9:1, *v*/*v*) and sequentially partitioned with solvents of increasing polarity to obtain hexane (HF), dichloromethane (DF), ethyl acetate (EAF), and n-butanol (BF) fractions. The remaining aqueous layer was designated as the aqueous fraction (AF). All fractions were concentrated using a rotary evaporator and lyophilized. For in vitro experiments, lyophilized extracts were dissolved in dimethyl sulfoxide (DMSO) and diluted to the desired concentrations. The ethyl acetate fraction (DMLEAF) was used for subsequent experiments.

### 2.3. Cell Culture and Cell Viability Assay

HepG2 cells were cultured in DMEM supplemented with 10% FBS, 100 U/mL penicillin, 100 μg/mL streptomycin, and 2 mM L-glutamine at 37 °C in a humidified incubator with 5% CO_2_. For cell viability assays, cells were seeded in 96-well plates at a density of 5 × 10^4^ cells/well and incubated for 24 h. To evaluate cytotoxicity of solvent fractions, cells were treated with various concentrations (12.5–200 μg/mL) of each fraction (HF, DF, EAF, BF, and AF) for 24 h. For ethanol-induced cytotoxicity experiments, cells were pretreated with DMLEAF at concentrations of 6.25–50 μg/mL for 1 h, followed by exposure to ethanol (400 mM) for 24 h. Cell viability was determined using the 3-(4,5-dimethylthiazol-2-yl)-2,5-diphenyltetrazolium bromide (MTT) assay. MTT solution (final concentration 1 mg/mL) was added and incubated for 4 h at 37 °C. The resulting formazan crystals were dissolved in DMSO, and absorbance was measured at 540 nm using a microplate reader (Molecular Devices, San Jose, CA, USA).

### 2.4. Measurement of Intracellular ROS

Intracellular ROS levels were measured using 2′,7′-dichlorofluorescein diacetate (DCFH-DA). HepG2 cells were seeded in black 96-well plates with clear bottoms at a density of 1 × 10^5^ cells/well. After treatment with DMLEAF for 24 h, ethanol was added. Six hours after ethanol treatment, cells were washed with DPBS and incubated with DCFH-DA (10 μM) for 30 min at 37 °C in the dark. Fluorescence intensity was measured at excitation/emission wavelengths of 485/530 nm.

### 2.5. Apoptosis Analysis

Apoptosis was evaluated by propidium iodide (PI) staining followed by flow cytometric analysis. HepG2 cells were seeded in 6-well plates at 5 × 10^5^ cells/well and treated with DMLEAF followed by ethanol exposure. After 24 h, cells were harvested and stained with PI staining solution (Abcam, Cambridge, UK) according to the manufacturer’s instructions. The percentage of apoptotic (sub-G1) cells was analyzed using a FACS Cell Analyzer (Becton Dickinson, San Jose, CA, USA).

### 2.6. Animal Model of Acute Ethanol-Induced Liver Injury

Eight-week-old male ICR mice (25–30 g) were obtained from DBL (Eumsung, Korea). Mice were housed under controlled conditions (22 ± 2 °C, 50 ± 5% humidity, 12 h light/dark cycle) with free access to food and water. After one week of acclimatization, mice were randomly divided into four groups (*n* = 6 per group). DMLEAF was dissolved in distilled water and orally administered by gavage at doses of 100 and 300 mg/kg body weight once daily for 7 days. The control group received distilled water. Ethanol (40%, 5 g/kg body weight) was orally administered by gavage three times at 12 h intervals starting on day 8. Four hours after the final ethanol administration, mice were euthanized by CO_2_ inhalation. Blood samples were collected via cardiac puncture and centrifuged at 3000× *g* for 10 min to obtain serum. Liver tissues were immediately excised for further analysis.

### 2.7. Measurement of Serum Biochemical Parameters

Blood samples were centrifuged at 3000× *g* for 10 min to obtain serum. Serum alanine transaminase (ALT), aspartate transaminase (AST), and ethanol levels were measured using an automated biochemical analyzer (Hitachi 7180, Hitachi High-Technologies, Tokyo, Japan). Serum ethanol concentrations were determined using an ethanol assay kit (Megazyme, Bray, Ireland).

### 2.8. Measurement of Antioxidant Enzyme Activity

Antioxidant enzyme activities in HepG2 cells and liver tissues were measured following previously reported methods. Cells and liver tissues were homogenized in 50 mM phosphate buffer and centrifuged at 3000× *g* for 20 min at 4 °C. The supernatants were used for enzyme activity assays. SOD activity was determined using the method of McCord and Fridovich [24], and CAT activity was measured according to Aebi [25]. GPx and GR activities were measured based on the method described by Koneru et al. [26]. Protein concentrations were determined using a bicinchoninic acid (BCA) protein assay kit (Thermo Fisher Scientific, Waltham, MA, USA).

### 2.9. Histological Analysis

Liver tissues were fixed in 10% neutral buffered formalin, embedded in paraffin, and sectioned at 3 μm thickness. Sections were deparaffinized, rehydrated, and stained with hematoxylin and eosin (H&E). Images were captured using an Olympus DP-72 microscope (Olympus, Tokyo, Japan) at 200× and 400× magnification.

### 2.10. Western Blotting

Twenty-four hours after DMLEAF and ethanol treatment, HepG2 cells were collected and lysed using RIPA buffer. The lysates were centrifuged at 5000× *g* for 10 min at 4 °C, and the supernatants were collected. Protein concentrations were determined using a BCA protein assay kit (Thermo Fisher Scientific, Waltham, MA, USA). Equal amounts of protein (30 μg) were separated by SDS–PAGE and transferred onto nitrocellulose membranes. The membranes were blocked with 5% bovine serum albumin (BSA) for 1 h at room temperature and then incubated overnight at 4 °C with primary antibodies. After washing, the membranes were incubated with appropriate horseradish peroxidase (HRP)-conjugated secondary antibodies for 1 h at room temperature. Protein bands were visualized using enhanced chemiluminescence (ECL) reagents and detected using an imaging system (LAS-3000 mini, Fujifilm, Tokyo, Japan). Information on the antibodies used is provided in the Supplementary Materials.

### 2.11. Real Time Quantitative Polymerase Chain Reaction

Total RNA was extracted from HepG2 cells and liver tissues using RNAiso Plus (Takara Bio Inc., Shiga, Japan). cDNA was synthesized using M-MLV reverse transcriptase (Promega, Madison, WI, USA). RT-qPCR was performed using SYBR Green Master Mix (Applied Biosystems, Foster City, CA, USA) on a StepOnePlus™ Real-Time PCR System. The amplification conditions were 95 °C for 10 min, followed by 40 cycles of 95 °C for 15 s and 60 °C for 60 s. Gene expression levels of Nrf2, CAT, SOD, GPx, GR, and CYP2E1 were normalized to glyceraldehyde-3-phosphate dehydrogenase (GAPDH) and calculated using the 2^−ΔΔCt^ method. Primer sequences are listed in the Supplementary Materials.

### 2.12. UPLC-ESI-Q-TOF-MS/MS Analysis

Phytochemical analysis of DMLEAF was performed using an HPLC system (Agilent Technologies, Santa Clara, CA, USA) equipped with a photodiode array detector (PDA) and a Q-TOF mass spectrometer (Bruker Daltonics, Bremen, Germany), as previously described [23]. Separation was achieved using a reverse-phase C18 column (250 × 4.6 mm, 5 μm) with a gradient mobile phase consisting of water (0.1% TFA) and acetonitrile (0.1% TFA) at a flow rate of 1 mL/min. Detection wavelengths were set at 210, 316, 365, and 520 nm. Mass spectrometry analysis was conducted in positive ion mode over a mass range of *m*/*z* 50–1000 using an electrospray ionization (ESI) source. Detailed instrumental parameters followed the previously reported method [23].

### 2.13. Statistical Analysis

All data are expressed as mean ± standard deviation (SD). Statistical analysis was performed using one-way analysis of variance (ANOVA) followed by Tukey’s post hoc test for multiple comparisons. Prior to analysis, data were assessed for normality and homogeneity of variance. Statistical analyses were conducted using Minitab software (Version 17, Minitab Inc., State College, PA, USA). Differences were considered statistically significant at *p* < 0.05.

## 3. Results

### 3.1. DMLEAF Exhibits the Strongest Cytoprotective Activity Against Ethanol-Induced Hepatotoxicity

**Figure 1:**
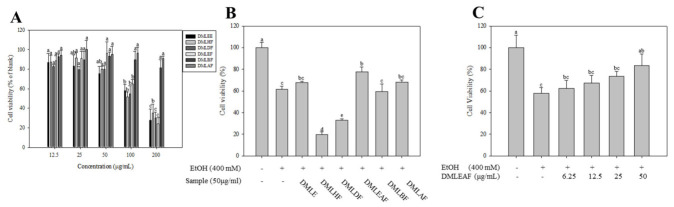
Cell viability and protective effects of *Dendropanax morbifera* solvent fraction and DMLEAF on ethanol induced cell damage. HepG2 cells were treated with various concentrations (12.5, 25, 50, 100, 200 μg/mL) of *D. morbifera* solvent fraction for 24 h. Cell viability was measured by MTT assay (**A**). HepG2 cells were treated with each solvent fraction of 50 μg/mL, and then treated with ethanol after 1 h. After 24 h of ethanol treatment, cell viability was measured using the MTT test method (**B**). HepG2 cells were treated with DMLEAF at various concentrations (6.25–50 μg/mL) and then treated with ethanol. After 24 h, cell viability was measured using the MTT assay (**C**). Values are mean ± SE (*n* = 3). Other letters above the bar indicate significant differences (by ANOVA, *p* < 0.05).

Prior to evaluating the hepatoprotective activity of each solvent fraction of *Dendropanax morbifera* leaves, cytotoxicity was assessed in HepG2 cells to determine non-toxic working concentrations (Figure 1A). The DMLBF and DMLAF did not exhibit cytotoxicity at any tested concentration (12.5–200 μg/mL). In contrast, the DMLEE, DMLHF, DMLDF, and ethyl DMLEAF showed no significant cytotoxicity at concentrations ranging from 12.5 to 50 μg/mL, but induced dose-dependent reductions in cell viability at 100 and 200 μg/mL. Based on these findings, 50 μg/mL was selected as the maximum non-cytotoxic concentration for subsequent cytoprotective assays.

To evaluate the protective effects against ethanol-induced hepatotoxicity, HepG2 cells were pretreated with each fraction (50 μg/mL) for 1 h, followed by exposure to 400 mM ethanol for 24 h, which reduced cell viability by approximately 42% compared to untreated controls (Figure 1B). Treatment with each fraction (50 μg/mL) revealed that DMLHF, DMLDF, and DMLBF did not significantly attenuate ethanol-induced cytotoxicity. In contrast, DMLEE and DMLAF showed a tendency to increase cell viability, whereas DMLEAF significantly improved cell viability compared with the ethanol-treated group. Among these, DMLEAF exhibited the most pronounced protective effect, restoring cell viability by 29.73% relative to ethanol-treated cells.

To further characterize the dose-dependent effects of DMLEAF, cells were pretreated with DMLEAF at concentrations of 12.5, 25, and 50 μg/mL for 1 h prior to ethanol exposure (400 mM, 24 h) (Figure 1C). DMLEAF treatment showed a concentration-related increase in cell viability, enhancing viability by 12.8%, 16.0%, and 27.2%, respectively, compared with ethanol-treated cells. A statistically significant protective effect was observed at 50 μg/mL. These results indicate that the ethyl acetate fraction of *D. morbifera* leaves exhibits the strongest protective effect against ethanol-induced cytotoxicity among the tested fractions and was therefore selected for subsequent phytochemical and mechanistic investigations.

### 3.2. DMLEAF Attenuates Ethanol-Induced Apoptosis and ROS Generation in HepG2 Cells

**Figure 2:**
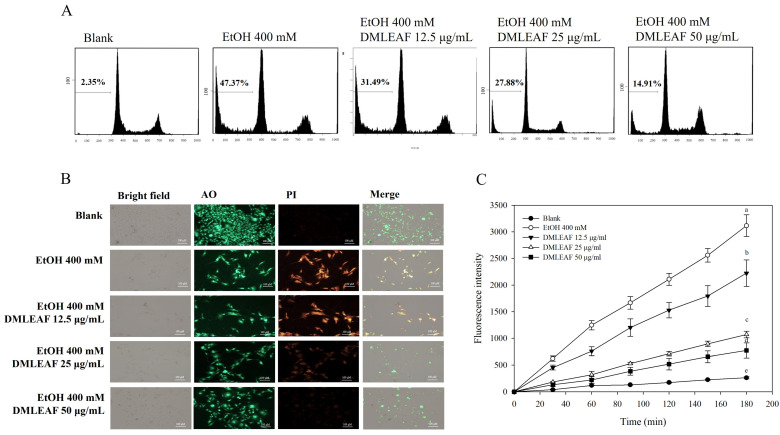
Effects of DMLEAF on ethanol induced apoptosis. HepG2 cells were treated with DMLEAF by concentration, and then treated with ethanol after 1 h. After 24 h, the cells were stained with PI and then apoptosis was analyzed using flow cytometry (**A**). Cell damage was induced by treatment with DMLEAF and ethanol, and then the cells in which apoptosis occurred were analyzed through AO/PI double staining (**B**). After treatment with DMLEAF and ethanol to induce ROS generation, the generated ROS was analyzed using DCFDA reagent Values are mean ± SE (*n* = 3). Other letters above the bar indicate significant differences (by ANOVA, *p* < 0.05) (**C**).

To determine whether the cytoprotective effect of the DMLEAF was associated with inhibition of apoptosis, ethanol-induced apoptotic cell death was evaluated by flow cytometric analysis following PI staining (Figure 2A). Exposure of HepG2 cells to 400 mM ethanol markedly increased the proportion of apoptotic cells to 47.37%, compared with untreated controls. Treatment with DMLEAF significantly reduced ethanol-induced apoptosis in a concentration-dependent manner. The percentage of apoptotic cells decreased to 31.49%, 27.88%, and 14.91% following treatment with 12.5, 25, and 50 μg/mL DMLEAF, respectively. These results indicate that DMLEAF effectively attenuates ethanol-induced apoptotic cell death.

Apoptotic morphology was further confirmed by acridine orange/propidium iodide (AO/PI) dual staining (Figure 2B). Control cells were predominantly stained with AO, indicating viable cells with intact membranes. In contrast, ethanol-treated cells exhibited increased PI-positive staining, reflecting membrane damage and apoptotic cell death. Notably, DMLEAF treatment markedly reduced the number of PI-positive cells, further supporting its anti-apoptotic effect.

Because ethanol-induced apoptosis is closely associated with oxidative stress, intracellular ROS generation was assessed using DCFH-DA fluorescence (Figure 2C). Ethanol exposure led to a rapid and sustained increase in intracellular ROS levels over time. However, pretreatment with DMLEAF significantly suppressed ethanol-induced ROS accumulation in a concentration-dependent manner. Fluorescence microscopy confirmed these findings, showing intense DCF fluorescence in ethanol-treated cells, whereas DMLEAF pretreated cells exhibited markedly reduced fluorescence intensity. Collectively, these results suggest that DMLEAF protects hepatocytes against ethanol-induced apoptosis primarily through suppression of intracellular ROS generation.

### 3.3. DMLEAF Attenuated Antioxidant Enzyme Activities and Protein Expression in Ethanol-Treated HepG2 Cells

**Figure 3:**
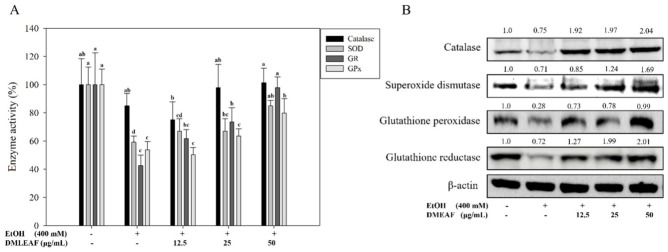
Effect of DMLEAF treatment on antioxidant enzyme activity in ethanol treated HepG2 cells. Values are mean ± SE (*n* = 3). Other letters above the bar indicate significant differences (by ANOVA, *p* < 0.05) (**A**). Protein expression analysis to examine the effect of DMLEAF on antioxidant enzyme in ethanol treated HepG2 cell (**B**).

To investigate whether DMLEAF modulates endogenous antioxidant defense systems impaired by ethanol exposure, the activities of major antioxidant enzymes were measured in HepG2 cells (Figure 3A). Ethanol treatment significantly reduced CAT activity by approximately 24% and SOD activity by approximately 41% compared with untreated controls, indicating marked oxidative stress. Treatment with DMLEAF at 12.5 μg/mL did not significantly restore CAT or SOD activities. However, 25 μg/mL DMLEAF increased CAT and SOD activities by 11% and 22%, respectively, compared with ethanol-treated cells. At 50 μg/mL, CAT activity increased by 15%, while SOD activity increased by 24%, demonstrating a concentration-dependent recovery of enzymatic antioxidant capacity. In addition, glutathione-related antioxidant enzymes were evaluated. Ethanol exposure significantly reduced the activities of GPx and GR, both of which are critical for maintaining intracellular redox balance through glutathione metabolism. Treatment with 25 and 50 μg/mL DMLEAF significantly restored GPx and GR activities, consistent with the recovery observed for CAT and SOD.

To determine whether these changes in enzymatic activity were associated with altered protein expression, Western blot analysis was performed (Figure 3B). Consistent with the activity assays, ethanol treatment reduced the protein expression levels of CAT, SOD, GPx, and GR. DMLEAF treatment at 25 and 50 μg/mL markedly increased the expression of these antioxidant enzymes relative to ethanol-treated cells. These findings suggest that DMLEAF attenuated antioxidant defense capacity at both functional and protein expression levels.

### 3.4. DMLEAF Activates Nrf2 Signaling and Promotes Nuclear Translocation in HepG2 Cells

**Figure 4:**
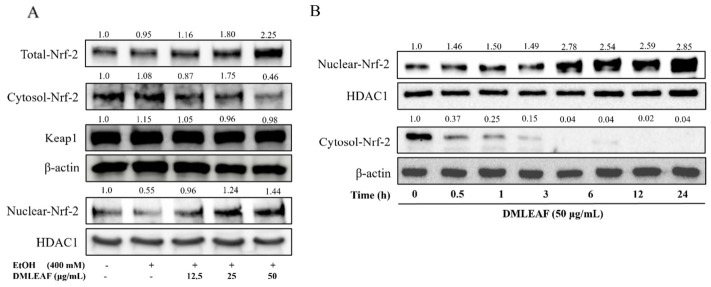
Effect of DMLEAF on Nrf2 translocation in ethanol-induced HepG2 cells. (**A**) HepG2 cells were pretreated with DMLEAF (12.5, 25, and 50 μg/mL) for 1 h and subsequently exposed to 400 mM ethanol for 24 h. Cytosolic and nuclear Nrf2 protein levels, as well as Keap1 expression, were analyzed by Western blotting. β-Actin and HDAC1 were used as loading controls for cytosolic and nuclear fractions, respectively. (**B**) HepG2 cells were pretreated with 50 μg/mL DMLEAF and then exposed to 400 mM ethanol. Nuclear and cytosolic Nrf2 protein levels were analyzed by Western blotting at the indicated time points (0–24 h) to evaluate the time-dependent translocation of Nrf2. HDAC1 and β-actin were used as loading controls for nuclear and cytosolic fractions, respectively. Relative band intensities are shown above each band.

Because the expression of antioxidant enzymes is primarily regulated by Nrf2, we next examined whether DMLEAF activates the Nrf2 signaling pathway (Figure 4). Under basal conditions, Nrf2 is retained in the cytoplasm through interaction with Keap1. Upon activation, Nrf2 dissociates from Keap1 and translocates into the nucleus to initiate transcription of ARE-dependent genes.

Western blot analysis revealed that total Nrf2 protein expression was increased following treatment with 25 and 50 μg/mL DMLEAF. Furthermore, subcellular fractionation demonstrated a marked increase in nuclear Nrf2 levels accompanied by a corresponding decrease in cytosolic Nrf2, indicating enhanced nuclear translocation (Figure 4A,B). In contrast, Keap1 protein expression remained unchanged following DMLEAF treatment. Time-course analysis further confirmed that DMLEAF induced progressive accumulation of Nrf2 in the nuclear fraction, supporting activation of Nrf2-dependent transcriptional signaling. These results suggest that DMLEAF enhances antioxidant enzyme expression and supports the involvement of Nrf2-mediated antioxidant signaling, thereby contributing to protection against ethanol-induced oxidative stress.

### 3.5. DMLEAF Attenuates Ethanol-Induced Liver Injury in Mice

**Figure 5:**
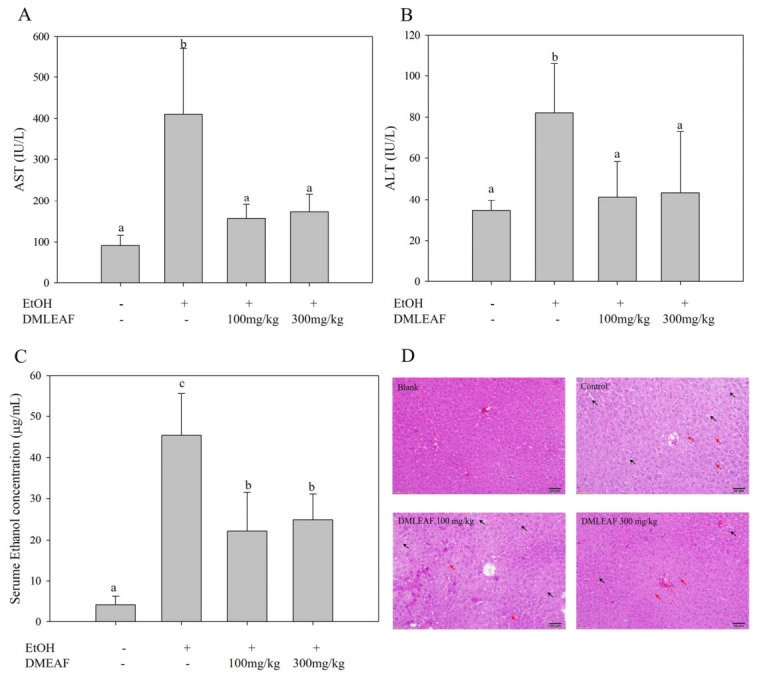
Hepatoprotective effects of DMLEAF in mice with acute ethanol-induced liver injury. Serum AST (**A**), ALT (**B**), and ethanol concentrations (**C**) were measured in mice. Data are expressed as mean ± SD (*n* = 6), and different letters indicate significant differences among groups (*p* < 0.05). Representative H&E-stained liver sections are shown (**D**). The ethanol-treated control group exhibited marked histopathological alterations, including hepatocyte ballooning and inflammatory cell infiltration. In contrast, DMLEAF-treated groups (100 and 300 mg/kg) showed attenuated hepatic damage and improved cellular architecture. Black arrows indicate hepatocyte ballooning and structural damage, whereas red arrows indicate inflammatory cell infiltration.

To validate the hepatoprotective effects observed in HepG2 cells, an acute ethanol-induced liver injury mouse model was employed (Figure 5). Mice were orally administered DMLEAF (100 or 300 mg/kg) for seven consecutive days, followed by high-dose ethanol exposure. Animals were sacrificed 8 h after the final ethanol administration, and serum biochemical markers and liver tissues were analyzed. Serum ALT and AST activities were significantly elevated in the ethanol-treated group compared with the normal control group (*p* < 0.05), indicating hepatic injury (Figure 5A,B). Oral administration of DMLEAF markedly attenuated ethanol-induced increases in ALT and AST levels. Both 100 and 300 mg/kg DMLEAF reduced serum transaminase activities by more than two-fold relative to the ethanol group, although no significant difference was observed between the two doses. Serum ethanol concentration was also measured as an indirect indicator of hepatic ethanol metabolism (Figure 5C). Ethanol-treated mice exhibited sustained elevation of blood ethanol levels. In contrast, DMLEAF administration significantly reduced serum ethanol concentrations compared with the ethanol-only group, suggesting improved ethanol clearance.

Histopathological examination of liver tissues using H&E staining revealed normal hepatic architecture in the control group. Ethanol exposure induced hepatocellular hypertrophy, disrupted cellular organization, and structural damage. Notably, DMLEAF treatment ameliorated these pathological alterations, restoring hepatic architecture and reducing hepatocellular swelling (Figure 5D). These findings demonstrate that DMLEAF exerts significant hepatoprotective effects in vivo.

### 3.6. DMLEAF Restores Hepatic Antioxidant Enzyme Activities in Ethanol-Treated Mice

**Figure 6:**
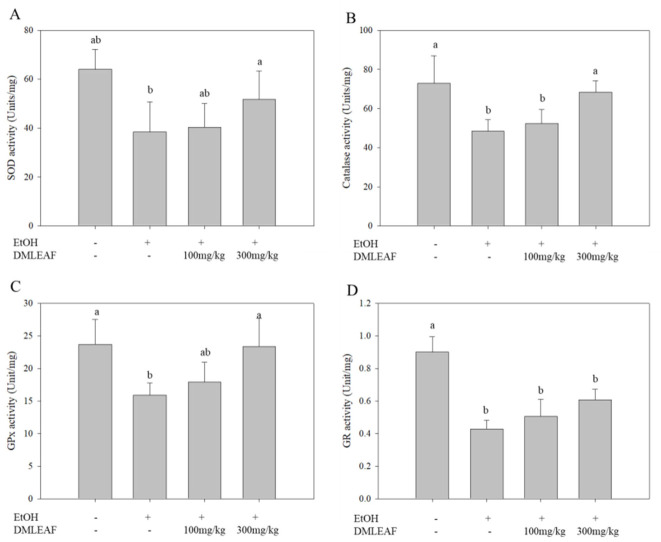
Effects of the DMLEAF on antioxidant enzyme activity in acute alcohol induced liver damage. (**A**) SOD activity, (**B**) Catalase activity, (**C**) GPx activity and (**D**) GR activity. The data are expressed as the mean ± SD (*n* = 6), and different letters indicate a significant difference at *p* < 0.05, as determined by ANOVA.

To determine whether the hepatoprotective effects of DMLEAF were associated with modulation of oxidative stress, hepatic antioxidant enzyme activities were measured (Figure 6). Ethanol administration significantly decreased SOD activity compared with the normal group (*p* < 0.05). DMLEAF treatment significantly attenuated SOD activity at both tested doses (*p* < 0.001 vs. ethanol group) (Figure 6A).

CAT activity showed a modest reduction following ethanol exposure. Although DMLEAF administration increased CAT activity, the effect was less pronounced than that observed for SOD (Figure 6B). Similarly, glutathione-dependent enzymes, including GR and GPx, were slightly decreased by ethanol treatment. Administration of high-dose DMLEAF (300 mg/kg) significantly restored GR and GPx activities compared with the ethanol-treated group, whereas the low-dose group exhibited moderate effects (Figure 6C,D). These results indicate that DMLEAF enhances hepatic antioxidant defense systems in vivo, consistent with the findings observed in HepG2 cells.

### 3.7. DMLEAF Modulates Hepatic Gene Expression of CYP2E1 and Antioxidant Enzymes

**Figure 7:**
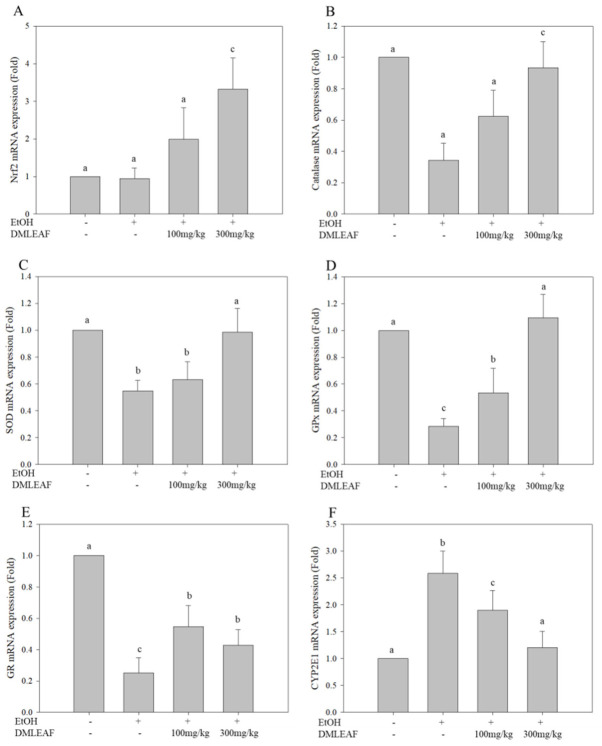
Effects of the DMLEAF on Nrf2, antioxidant enzyme, CYP2E1 gene expression in acute alcohol induced liver damage. Nrf2 (**A**), Catalase (**B**), SOD (**C**), GPx (**D**), GR (**E**) and CYP2E1 (**F**) expression. The data are expressed as the mean ± SD (*n* = 6), and different letters indicate a significant difference at *p* < 0.05, as determined by ANOVA.

To assess whether alterations in enzyme activity were associated with transcriptional regulation, hepatic mRNA expression levels were analyzed by real-time quantitative PCR (Figure 7). Ethanol administration significantly increased CYP2E1 mRNA expression by approximately 2.6-fold compared with the control group (Figure 7F). DMLEAF treatment significantly suppressed ethanol-induced CYP2E1 upregulation. Consistent with enzyme activity results, the mRNA expression levels of antioxidant enzymes—including CAT, SOD1, GPx, and GR—were markedly reduced in the ethanol-treated group. While 100 mg/kg DMLEAF did not produce statistically significant changes, administration of 300 mg/kg DMLEAF significantly upregulated the transcription of these antioxidant genes compared with the ethanol group (Figure 7B–E). These findings suggest that DMLEAF regulates hepatic redox homeostasis through transcriptional modulation of CYP2E1 and Nrf2-target antioxidant genes.

### 3.8. Phytochemical Characterization of DMLEAF by UPLC-ESI-Q-TOF-MS/MS

According to previous studies, *Dendropanax morbifera* leaf contains various polyphenolic compounds, mainly flavonoids, flavonoid glycosides, and phenolic acid derivatives. To identify compounds potentially responsible for the protective effects against alcoholic liver injury, the polyphenolic constituents of DMLEAF were analyzed using HPLC–Q-TOF–MS/MS. The HPLC UV chromatograms (280 nm) and total ion chromatograms of DMLEAF are presented in Supplementary Figures S1–S3. Table 1 summarizes the identified compounds based on their deprotonated molecular ions observed in negative ion mode, MS/MS fragment ions, and corresponding MS data.

The Q-TOF MS/MS analysis revealed that the major flavonoid compounds in DMLEAF included quercetin glycosides such as rutin (quercetin-O-rutinoside), isoquercitrin (quercetin-O-glucoside), and kaempferol-O-rutinoside. In addition, luteolin-C-glycoside was also identified based on its characteristic fragmentation behavior.

At retention times of 11.9 and 12.9 min, deprotonated molecular ion peaks at *m*/*z* 447 [M–H]^−^ were observed. The MS/MS spectra of both peaks exhibited characteristic fragment ions at *m*/*z* 327 and 357, corresponding to neutral losses of 120 and 90 Da, respectively, which are indicative of cross-ring cleavage of the sugar moiety in flavonoid C-glycosides. In addition, fragment ions at *m*/*z* 285, corresponding to the luteolin aglycone, as well as diagnostic flavonoid ring cleavage ions at *m*/*z* 151 and 133, were detected. Based on these fragmentation patterns, both compounds were tentatively identified as luteolin-C-glucoside isomers. Furthermore, the earlier eluting peak at 11.9 min was assigned as luteolin-6-C-glucoside (isoorientin), while the later eluting peak at 12.9 min was assigned as luteolin-8-C-glucoside (orientin), based on their retention behavior in reversed-phase LC and the relative intensities of fragment ions. Specifically, the fragment ion at *m*/*z* 357 (*m*/*z* –90) was more prominent in the earlier peak, whereas the fragment ion at *m*/*z* 327 (*m*/*z* –120) showed higher intensity in the later peak.

At retention times of 13.2 and 13.6 min, deprotonated molecular ions at *m*/*z* 609 [M–H]^−^ were observed. The MS/MS spectra showed a prominent fragment ion at *m*/*z* 301, corresponding to the quercetin aglycone, generated by the neutral loss of a rutinoside moiety (*m*/*z* 308; rhamnose + glucose). These results indicate that rhamnose and glucose are conjugated to the flavonoid structure. Based on previous studies, quercetin-7-O-rutinoside is more hydrophilic and elutes earlier than quercetin-3-O-rutinoside in reversed-phase LC systems. Therefore, the peaks at 13.2 and 13.6 min were tentatively assigned as quercetin-7-O-rutinoside and quercetin-3-O-rutinoside, respectively.

At a retention time of 16.5 min, a deprotonated molecular ion peak at *m*/*z* 593.14 [M–H]^−^ was observed. The MS/MS spectrum showed a fragment ion at *m*/*z* 285.03, corresponding to the loss of a rutinoside moiety (*m*/*z* 308), indicating the presence of a flavonoid glycoside. The aglycone ion at *m*/*z* 285.03 was identified as kaempferol (molecular weight 286). Accordingly, this compound was tentatively identified as kaempferol-O-rutinoside.

At retention times of 14.2, 17.8, and 19.1 min, deprotonated molecular ion peaks at *m/z* 463.08 [M–H]^−^ were observed, indicating the presence of quercetin-O-hexoside isomers. The MS/MS spectra of all three peaks showed a prominent fragment ion at *m/z* 301, corresponding to the neutral loss of 162 Da, which is characteristic of the cleavage of a hexose moiety. The fragment ion at *m/z* 301 was assigned to the quercetin aglycone. Based on their retention behavior in reversed-phase LC, the peaks were tentatively assigned as Hyperoside (14.2 min), Isoquercitrin (17.8 min), and Quercetin-7-O-glucoside (19.1 min), consistent with the typical elution order (3-O-galactoside < 3-O-glucoside < 7-O-glucoside). However, due to the identical exact masses and highly similar MS/MS fragmentation patterns of these isomers, definitive discrimination—particularly between galactoside and glucoside forms—cannot be achieved by qTOF-MS alone, and the assignments are therefore primarily based on chromatographic retention behavior.

Three peaks exhibiting identical deprotonated molecular ions at *m/z* 353 [M–H]^−^ were detected at retention times of 5.6, 6.7, and 7.2 min, indicating the presence of caffeoylquinic acid (CQA) isomers. MS/MS analysis revealed that the peak at 5.6 min produced characteristic fragment ions at *m/z* 191 (quinic acid) and *m/z* 179 (caffeic acid), allowing its assignment as 3-caffeoylquinic acid (3-CQA, neochlorogenic acid). The peak at 6.7 min showed a dominant fragment ion at *m/z* 191 with negligible *m/z* 179, which is consistent with 5-caffeoylquinic acid (5-CQA, chlorogenic acid). The peak at 7.2 min, eluting last among the isomers, was tentatively assigned as 4-caffeoylquinic acid (4-CQA, cryptochlorogenic acid) based on its retention behavior in reversed-phase LC. The observed elution order (3-CQA < 5-CQA < 4-CQA) and fragmentation patterns are in excellent agreement with previously reported data for CQA isomers.

Furthermore, peaks exhibiting identical deprotonated molecular ions at *m/z* 515.11 [M–H]^−^ were observed at retention times of 17.2 and 18.7 min, indicating dicaffeoylquinic acid (diCQA) isomers. The MS/MS spectra showed a prominent fragment ion at *m/z* 353, corresponding to the loss of one caffeoyl moiety (162 Da). For the peak at 17.2 min, the *m/z* 353 fragment further produced a dominant ion at *m/z* 191 with minor *m/z* 179, suggesting a 5-CQA-type substructure. This peak was therefore assigned as 3,5-dicaffeoylquinic acid, also known as isochlorogenic acid A. In contrast, the peak at 18.7 min exhibited relatively weaker *m/z* 353 intensity and produced fragment ions with a stronger *m/z* 179 and characteristic *m/z* 155, indicating the involvement of a 4-CQA-type substructure. Accordingly, this peak was tentatively assigned as 4,5-dicaffeoylquinic acid, also known as isochlorogenic acid C. These assignments are consistent with reported fragmentation behavior and retention characteristics of diCQA isomers in reversed-phase LC. These results indicate that DMLEAF is enriched in polyphenolic compounds, particularly chlorogenic acid and rutin, which are known to possess potent antioxidant properties and may contribute to its hepatoprotective effects.

## 4. Discussion

Alcohol-induced liver injury is primarily driven by excessive generation of ROS during ethanol metabolism, leading to oxidative stress, hepatocellular damage, and progression of liver dysfunction. While ADH and ALDH are responsible for the primary metabolism of ethanol, CYP2E1 becomes increasingly involved under conditions of high alcohol exposure, producing substantial amounts of ROS as metabolic by-products [4,5]. This CYP2E1-mediated oxidative burden is a key contributor to lipid peroxidation, mitochondrial dysfunction, and hepatocyte apoptosis [27,28]. Accordingly, modulation of redox homeostasis through both suppression of ROS generation and enhancement of endogenous antioxidant defenses represents a central therapeutic strategy for alcoholic liver disease [29,30,31].

*D. morbifera* has been widely investigated for its diverse bioactivities [17]. Previous studies, including our own, have demonstrated that polyphenol-rich extracts from its leaves enhance alcohol-metabolizing enzymes and attenuate oxidative liver damage [22,23,32]. In the present study, solvent fractionation was employed to enrich bioactive constituents from *D. morbifera* leaves based on polarity. Among the fractions obtained, the ethyl acetate fraction (DMLEAF) exhibited the most potent antioxidant and hepatoprotective activities and was therefore selected for further investigation. This observation is consistent with the preferential partitioning of polyphenolic compounds, particularly flavonoid glycosides and phenolic acid derivatives, into moderately polar solvents such as ethyl acetate [33,34,35]. The superior activity of DMLEAF compared with other fractions suggests that the enrichment of specific polyphenolic constituents plays a critical role in mediating its biological effects.

To elucidate the chemical basis underlying these activities, comprehensive phytochemical profiling of DMLEAF was performed using HPLC–ESI–Q-TOF–MS/MS. A total of 20 polyphenolic compounds were tentatively identified, predominantly comprising chlorogenic acid derivatives (including caffeoylquinic acids and dicaffeoylquinic acids) and flavonoid glycosides derived from quercetin, kaempferol, and luteolin. Notably, multiple isomeric forms of caffeoylquinic acids (3-, 4-, and 5-CQA) and dicaffeoylquinic acids, as well as quercetin-O-glycosides and luteolin-C-glycosides, were detected, indicating a structurally diverse and polyphenol-rich composition. These compound classes are well documented for their potent antioxidant and hepatoprotective properties [36,37,38]. Chlorogenic acid derivatives have been reported to attenuate alcohol-induced oxidative stress and liver injury, and their hepatoprotective effects have been associated with modulation of CYP2E1-mediated ROS generation and activation of Nrf2-dependent antioxidant signaling pathways [39,40]. Furthermore, flavonoid glycosides identified in DMLEAF, including rutin, isoquercitrin, and luteolin derivatives, have been widely reported to enhance cellular antioxidant defenses through ROS scavenging and regulation of Nrf2-associated pathways, while simultaneously suppressing pro-inflammatory signaling cascades [41,42,43]. Therefore, the enrichment of these bioactive polyphenols in DMLEAF provides a biochemical basis for its superior efficacy among the tested fractions.

Consistent with this chemical composition, DMLEAF exerted strong antioxidant effects at the cellular level. Ethanol exposure markedly increased intracellular ROS generation and induced apoptotic cell death in HepG2 cells, whereas DMLEAF significantly attenuated ROS accumulation and reduced apoptosis. These findings indicate that the cytoprotective effects of DMLEAF extend beyond simple radical scavenging and involve restoration of intracellular redox balance. Mechanistically, DMLEAF enhanced both the activity and protein expression of key antioxidant enzymes, including SOD, CAT, GPx, and GR, which are essential components of the cellular antioxidant defense system. Restoration of these enzymatic pathways suggests that DMLEAF reinforces endogenous antioxidant capacity under oxidative stress conditions.

Importantly, the regulation of these antioxidant systems appears to be associated, at least in part, with the involvement of Nrf2-mediated antioxidant signaling [7,8,9]. Nrf2 is a master transcriptional regulator of antioxidant and cytoprotective genes, controlling the expression of enzymes involved in redox homeostasis [44,45]. In this study, DMLEAF increased total Nrf2 expression and promoted its nuclear translocation without significantly altering Keap1 protein levels, suggesting activation of Nrf2 through redox-sensitive mechanisms rather than changes in Keap1 abundance. Polyphenolic compounds are known to activate Nrf2 signaling through modification of cysteine residues in Keap1 or through indirect modulation of cellular redox status [46,47]. Given the abundance of chlorogenic acid derivatives and flavonoid glycosides identified in DMLEAF, it is plausible that these compounds act synergistically to facilitate Nrf2 activation, thereby upregulating downstream antioxidant defenses [42,43,48,49]. This chemical–mechanistic linkage provides a coherent explanation for the observed enhancement of antioxidant enzyme systems.

The hepatoprotective effects of DMLEAF were further confirmed in an acute ethanol-induced mouse model. Ethanol administration resulted in significant elevations in serum ALT and AST levels, indicative of hepatocellular injury, whereas DMLEAF treatment markedly reduced these biomarkers. Histopathological analysis corroborated these findings, demonstrating that DMLEAF alleviated hepatocellular hypertrophy and structural disruption induced by ethanol exposure. In addition, DMLEAF significantly decreased circulating ethanol levels, suggesting improved hepatic metabolic capacity and reduced systemic ethanol burden.

At the molecular level, ethanol-induced upregulation of hepatic CYP2E1 expression was significantly suppressed by DMLEAF. Given that CYP2E1 is a major source of ethanol-derived ROS [4,5], its downregulation, together with activation of Nrf2-dependent antioxidant pathways, indicates that DMLEAF mitigates oxidative stress through dual modulation of ROS production and detoxification systems. This coordinated regulation of oxidative stress pathways highlights the multifaceted mechanism underlying the hepatoprotective effects of DMLEAF.

Despite these findings, several limitations should be acknowledged. Although a diverse array of polyphenolic compounds was identified, most assignments were tentative and based on MS/MS fragmentation patterns without validation using authentic standards. In addition, while the enrichment of specific polyphenols in the ethyl acetate fraction was demonstrated, direct causal relationships between individual compounds and observed biological activities were not established. Furthermore, the in vitro experiments were conducted using HepG2 cells, which are widely used for studies of hepatic oxidative stress but possess lower ethanol-metabolizing capacity than primary hepatocytes. Therefore, the cellular responses observed in this study may not fully recapitulate the complexity of ethanol metabolism occurring in vivo. Moreover, although Nrf2 activation was observed, pathway dependency was not confirmed using genetic or pharmacological inhibition approaches. Future studies employing targeted compound isolation, quantitative analysis, and mechanistic validation using Nrf2-deficient models or specific inhibitors are warranted. Investigation under chronic alcohol exposure conditions and the use of primary hepatocytes or advanced liver models would also provide additional insight into the long-term therapeutic potential of DMLEAF.

In conclusion, this study demonstrates that the ethyl acetate fraction of *D. morbifera* leaves, enriched in chlorogenic acid derivatives and flavonoid glycosides, exerts potent hepatoprotective effects against alcohol-induced liver injury. These effects are mediated through coordinated suppression of CYP2E1-associated ROS generation and activation of Nrf2-dependent antioxidant defense systems. The integration of comprehensive phytochemical profiling with biological evaluation provides mechanistic insight into the functional role of plant-derived polyphenols and supports the potential application of DMLEAF as a natural therapeutic agent for alcoholic liver disease.

## 5. Conclusions

In the present study, the ethyl acetate fraction of *D. morbifera* leaves demonstrated significant antioxidant and hepatoprotective effects against ethanol-induced oxidative liver injury in both HepG2 cells and an acute mouse model. DMLEAF attenuated intracellular ROS accumulation and hepatocyte apoptosis and improved ethanol-induced alterations in liver function and histopathological features. In addition, DMLEAF mitigated the ethanol-induced impairment of antioxidant enzyme activity and expression. Mechanistically, these effects were associated with modulation of CYP2E1-related oxidative stress and activation of Nrf2-mediated antioxidant defense pathways, suggesting a role in maintaining cellular redox homeostasis. The hepatoprotective effects observed in this study are considered to arise from the overall bioactivity of the fraction rather than from specific individual constituents.

These findings support the potential of *D. morbifera*-derived fractions as functional bioactive agents for mitigating alcohol-induced liver injury. However, further studies are required to clarify the contribution of individual compounds and to validate the underlying mechanisms in more advanced experimental systems.

## Figures and Tables

**Table 1:** Identified phenolic components in *D. morbidera* leaf ethyl acetate fraction using UPLC Q-TOF analysis.

NO.	Proposed Compound	Molecular Formula	Rt (min)	Molecular Weight (Calc.)	[M–H]^−^ (Exp.)	MS Fragment
1	Protocatechuic acid	C_7_H_6_O_4_	4.9	154.11	153.01	109.02
2	Caffeoylquinic acid	C_16_H_18_O_9_	5.6	354.30	353.08	191.05, 173.04, 135.04
3	p-hydroxybenzoic acid	C_7_H_6_O_3_	6.1	138.12	137.02	
4	Caffeoylquinic acid	C_16_H_18_O_9_	6.7	354.30	353.08	191.05, 133.02, 127.09
5	Caffeoylquinic acid	C_16_H_18_O_9_	7.2	354.30	353.08	191.05, 173.04, 134.04
6	Caffeic acid	C_9_H_8_O_4_	7.5	180.16	179.03	135.04
7	Coumaroylquinic acid	C_16_H_18_O_8_	8.7	338.31	337.09	191.05, 173.04, 134.04, 109.02
8	Feruloylquinic acid	C_17_H_20_O_9_	10.5	368.3	367.05	191.05, 173.04, 134.03
9	Luteolin-C-glucoside	C_21_H_20_O_11_	11.9	448.4	447.09	327.04, 173.08, 163.00, 129.09
10	Luteolin-C-glucoside	C_21_H_20_O_11_	12.2	448.38	447.09	327.04, 290, 133
11	Quercetin-7-O-rutinoside	C_27_H_30_O_16_	13.2	610.51	609.14	301.03, 151.00
12	Isorhamnetin-C-glucoside	C_22_H_22_O_12_	13.4	432.40	431.09	341.06, 311.05, 283.05, 191.03, 161.02
13	Quercetin-3-O-rutinoside	C_27_H_30_O_16_	13.6	610.51	609.14	431.09, 300.02, 162.96, 151.00
14	Quercetin-O-glucoside	C_21_H_20_O_12_	14.2	464.38	463.08	300.02, 280, 178.99, 151.00, 134.04
15	Kaempferol-O-rutinoside	C_27_H_30_O_14_	16.5	594.52	593.14	327.04, 284.02, 258, 230
16	Dicaffeoylqunic acid	C_25_H_24_O_12_	17.2	516.45	515.11	353.08, 191.05, 187.09, 133.02, 125.09
17	Isorhamnetin-O-glycoside	C_22_H_22_O_12_	17.6	478.41	477.09	314.03, 271.02, 241.07, 105.56
18	Quercetin-O-glucoside	C_20_H_20_O_11_	17.8	464.38	463.08	293.03, 271.05, 151.00
19	Dicaffeoylqunic acid	C_25_H_24_O_12_	18.7	516.45	515.11	353.08, 191.05, 187.09, 133.02, 125.09
20	Quercetin-O-glucoside	C_21_H_20_O_12_	19.1	464.38	463.08	301.03, 227.05, 141.13, 136.98
21	Quercetin	C_25_H_24_O_12_	22.9		301.03	245.04, 178.99, 151.00, 134.04

## Data Availability

The data presented in this study are available from the corresponding author upon reasonable request.

## Supplementary Materials

The following supporting information can be downloaded at: https://www.mdpi.com/article/10.3390/nu18121902/s1, Figure S1: UV chromatogram and total ion chromatograms of DMLEAF obtained by UPLC-ESI-QTOF-MS in negative ion mode; Figure S2: MS/MS spectra of flavonoid compounds identified in DMLEAF. Figure S3: MS/MS spectra of phenolic acid compounds identified in DMLEAF. Table S1: Primary and secondary antibodies used for Western blot analysis. Table S2: Primer sequences used for real-time qPCR.
