# Supplementary material for: Dietary Polyphenols from Dendropanax morbifera Leaves Ameliorate Alcohol-Induced Liver Injury Through Regulation of Oxidative Stress and Nrf2 Signaling
Source: Nutrients. 2026 Jun 12;18(12):1902. doi: 10.3390/nu18121902 (PMC13306125; doi:10.3390/nu18121902)
Supplement: Supplementary file 1 [file nutrients-18-01902-s001.zip › nutrients-4339493-supplementary.pdf]

Supplementary Figure Legends

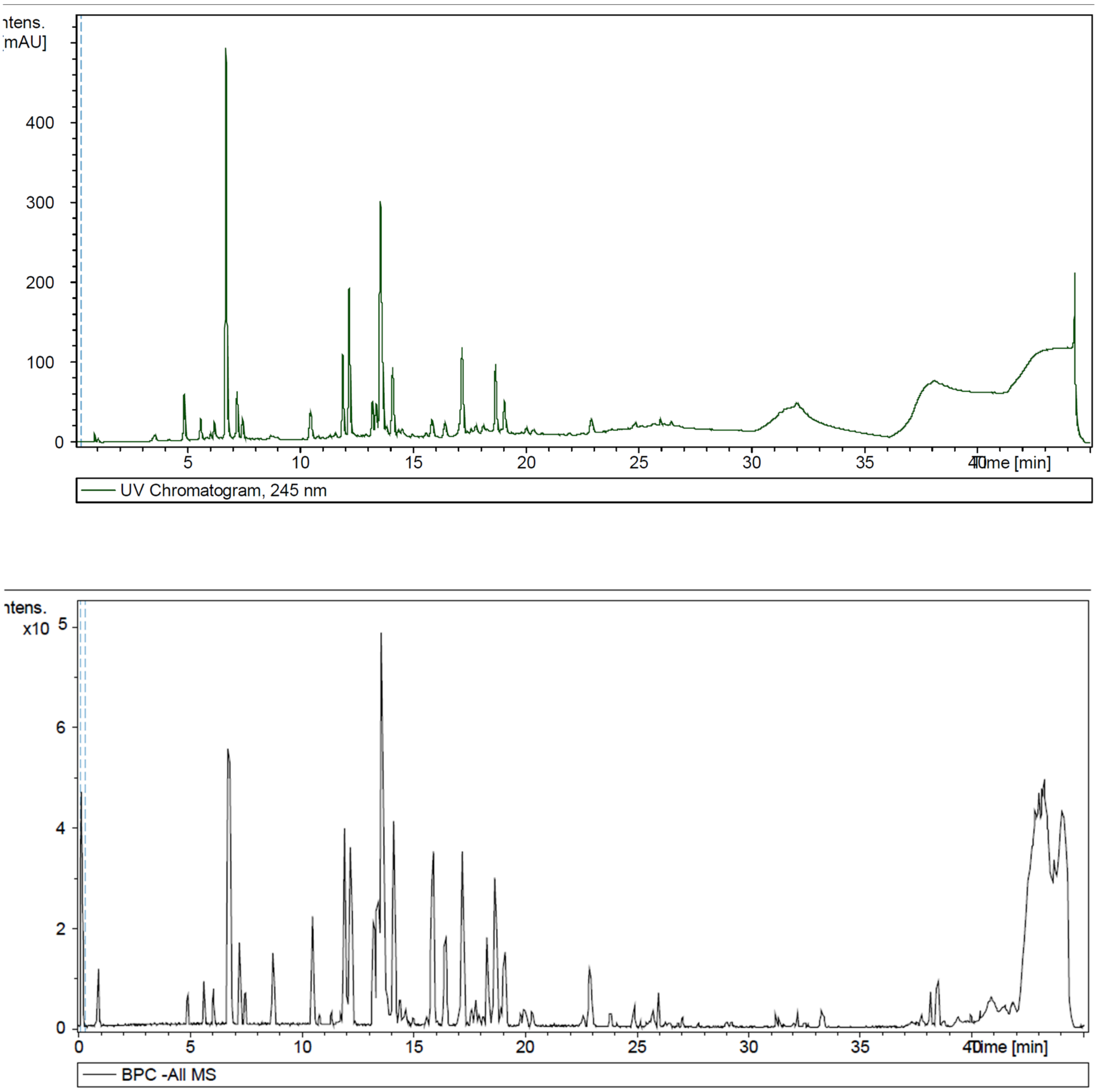

Figure S1 UV chromatogram and total ion chromatograms of DMLEAF obtained by UPLC–ESI–QTOF–MS in negative ion mode.

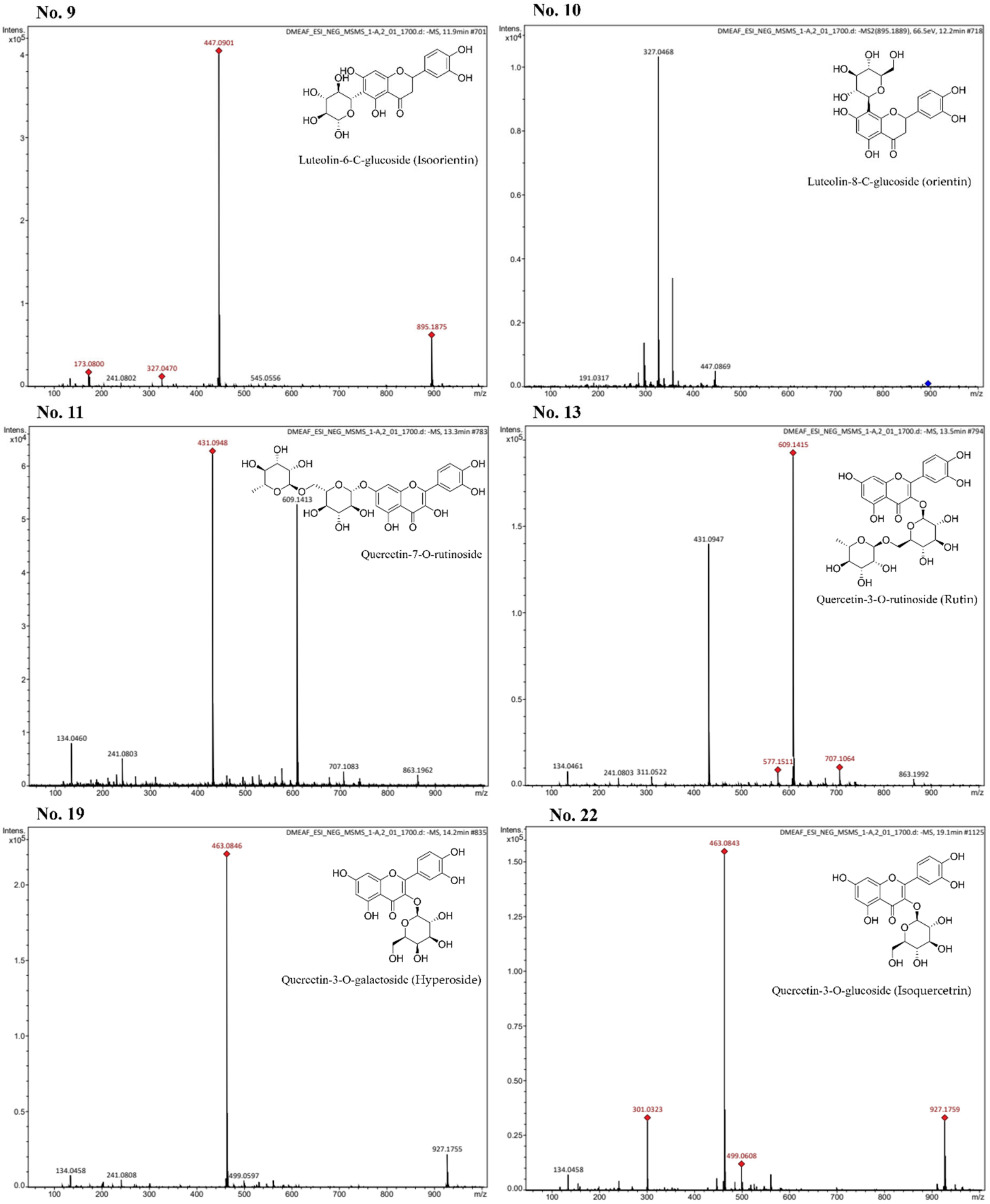

Figure S2 MS/MS spectra of flavonoid compounds identified in DMLEAF.

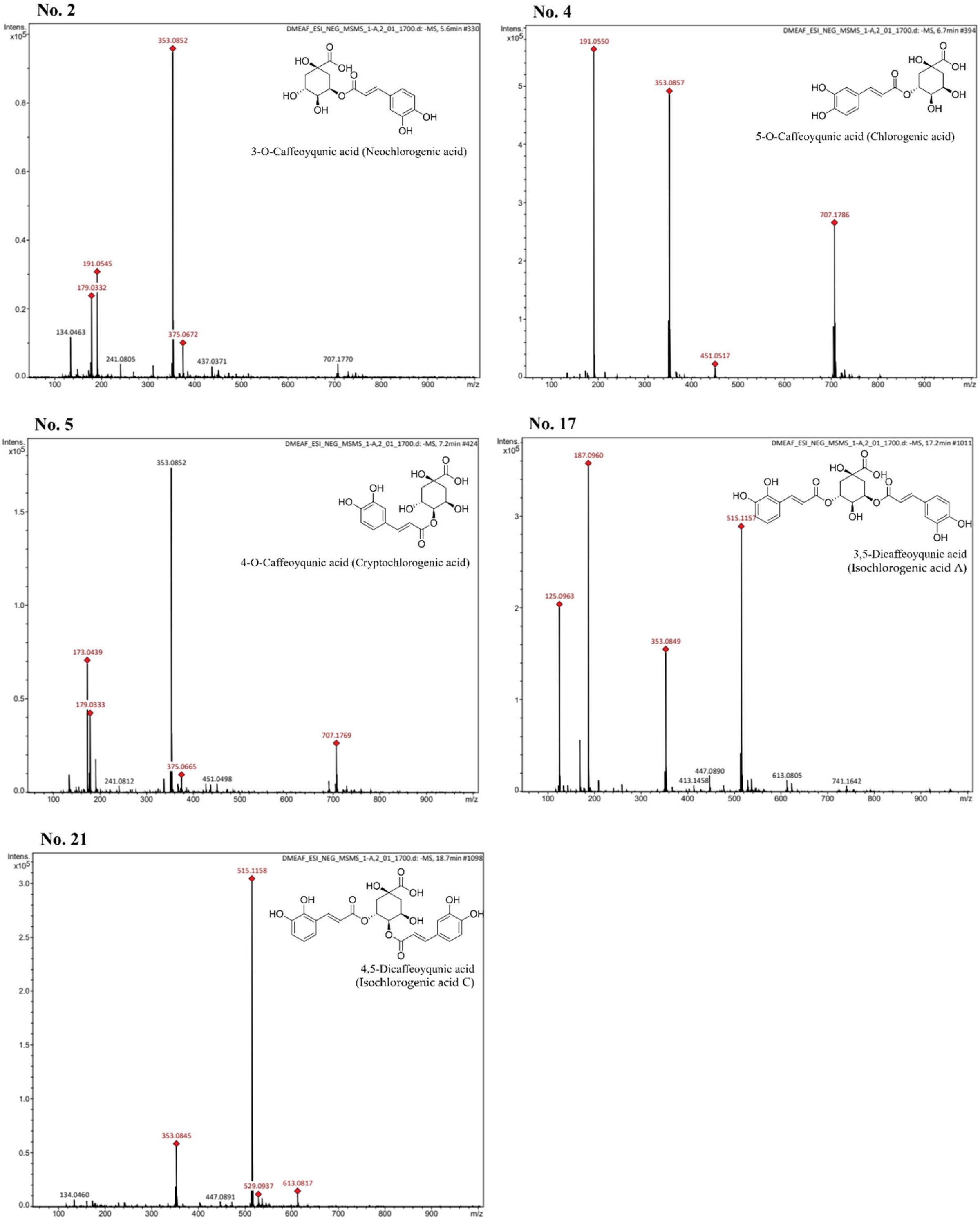

Figure S3 MS/MS spectra of phenolic acid compounds identified in DMLEAF.

Supplementary Table Legends

Table S1. Primary and secondary antibodies used for Western blot analysis.

Target Protein	Host	Dilution	Supplier	Number
Catalase	Mouse	1:1,000	Santacruz	sc-271803
Gpx	Mouse	1:1,000	Santacruz	sc-133160
GR	Mouse	1:1,000	Santacruz	sc-133245
SOD	Mouse	1:1,000	Santacruz	sc-101523
Nrf2	Rabbit	1:1,000	Cell signaling	#20733
Keap1	Mouse	1:1,000	Santacruz	sc-365626
β-Actin	Mouse	1:1,1000	Santacruz	sc-47778
HDAC	Rabbit	1:1,1000	Cell signaling	#34589
Anti-Rabbit IgG (HRP)	Goat	1:10, 000	Cell signaling	#7074
Anti-Mouse IgG (HRP)	Horse	1:10, 000	Cell signaling	#7076

Table S2. Primer sequences used for real-time qPCR.

Gene	Accession Number	Primer sequences
Nrf2	NM_008361	F: 5'-CATCAGGCCCAGTCCCTCAA-3'<br>R: 5'-AACAGCGGTAGATCAGCCA -3'
Catalase	NM_031168	F: 5'-GACAGGAAACGCCTGTGTGA-3'<br>R: 5'-CAGGGTGGACGTCAGTGAAA -3'
SOD1	NM_013693	F: 5'- TAACTGAAGGCCAGCATGGG-3'<br>R: 5'-ACATGCCTCTCTTCATCCGC-3'
GPx	NM_011198	F: 5'-CGTGCAATCAGTTCGGACAC -3'<br>R: 5'-TAAAGAGCGGGTGAGCCTTC-3'
GR	NM_010927	F: 5'-GCCGGAAACTTGCCCATAGAC-3'<br>R: 5'-ATACATCGGGGTAAAGGCAGTC-3'
CYP2E1	NM_010902	F: 5′-CCTTGCTTGTCTGGATCGCC-3′<br>R: 5′-AGTGTGAACACTGGCCCGAAG-3′
Actin	NM_001101	F: 5′-GGTGGGAATGGGTCAGAAGG-3′<br>R: 5′-CAGCACAGGGTGCTCCTC-3′
